# The complete chloroplast genome sequence of *Sloanea sinensis*

**DOI:** 10.1080/23802359.2021.1872453

**Published:** 2021-02-15

**Authors:** Yuhao Weng, Daiquan Ye, Yunfei You, Yitang Chen, Fujin Fan, Jisen Shi, Jinhui Chen

**Affiliations:** a Key Laboratory of Forest Genetics & Biotechnology of Ministry of Education of China, Co-Innovation Center for Sustainable Forestry in Southern China, Nanjing Forestry University, Nanjing, China; b National Germplasm Bank of Chinese fir at Fujian Yangkou Forest Farm, Shunchang, China

**Keywords:** Chloroplast genome, Elaeocarpaceae, *Sloanea sinensis*

## Abstract

*Sloanea sinensis* (Hance) Hu is a tree species and member of the Elaeocarpaceae family. It’s an excellent commercial tree species which has a relatively high net growth as forests. Here, we report the complete chloroplast genome sequence of a *Sloanea* genus for the first time. The complete chloroplast sequence of *S. sinensis* is 158,001 bp in length, including a large single copy region (LSC: 88,481 bp) and a small single copy region (SSC: 17,481 bp), the latter of which is separated by a pair of inverted repeat regions (IRs: 26,051 bp). Phylogenetic analysis indicates that the Elaeocarpaceae is a family within the Oxalidales may be more appropriate than belongs to Malvales as traditional plant taxonomy.

*Sloanea sinensis* (Hance) Hu is an evergreen tree species that belongs to the *Sloanea* genus, one of two genera that make up the Elaeocarpaceae. *S. sinensis* is widely distributed in southeastern China and part of southeast Asia, often growing in valleys or near streams. It is an excellent commercial tree species, providing tough, hard and heavy wood suitable for construction, furniture manufacturing etc. Until now, the only DNA sequence data that has been gathered on *Sloanea* is an incomplete chloroplast sequence from *Sloanea australis* (Foster et al. 2017). Complete sequencing of the chloroplast genome of *S. sinensis* may lead to a more thorough understanding of *Sloanea* photosynthesis and resulting wood production.

To obtain *S. sinensis* total chloroplast DNA, we collected leaf samples from the Yangkou Forest Farm, in Fujian province, China, located at 117.3 O–l18.14E, 26.39–27.12 N (voucher C2020HHX, deposited at Key Laboratory of Forest Genetics & Biotechnology of Ministry of Education of China Nanjing Forestry University). We extracted total DNA and used a HiSeq Xten platform with the PE150 strategy, run by Novogene (Nanjing), for sequencing and generated ∼5.61 G of raw reads. After filtering using SAMtools (Li 2011) and Fastp (Chen et al. 2018), we obtained ∼5.58 G of clean read data. We used the software package velvet (1.2.10) (Zerbino et al. 2009) to assemble the chloroplast genome. We then downloaded reference sequences from NCBI (GenBank accession: NC_044468, NC_039569, NC_037242) and used Geneious Prime (v2020.2.4) and tRNA-SCAN (Chan and Lowe 2019) to manually annotate the genomic sequences. Finally, we uploaded the complete *S. sinensis* chloroplast genome sequence, including annotation, to GenBank filed under accession number MW190090.

Analysis of the complete *S. sinensis* chloroplast genome sequence, which we found to be 158,001 bp in length, revealed a typical quadripartite construction: a large single copy region (LSC) of 88,481 bp in length, a small single copy region (SSC) of 17,481 bp in length and two inverted repeat regions (IRs), each 26,051 bp in length. The total GC content is ∼37.2%, while the GC content of the LSC, SSC and IRs regions is ∼42.8%, ∼31.5% and ∼35.0%, respectively. We annotated 101 unique genes, including 68 protein-coding genes, 29 tRNA genes and 4 rRNA genes. Because a small number of sequences contained genic mutations leading to premature STOP codons that would disrupt the open reading frame (ORF), we annotated these to be incomplete or pseudogenes. Therefore, the number of functional genes in the *S. sinensis* chloroplast genome appears to be smaller than in species to which it is directly related.

To better understand the phylogenetic relationship of *S. sinensis* to these direct relatives, we generated a multiple chloroplast genome sequence alignment using Clustal Omega (Sievers and Higgins 2018) and from this constructed a phylogenetic tree using MrBayes (default parameters, 50,000 generations) (Ronquist et al. 2012). For our analysis, we used 36 plant species, including *S. sinensis* itself and 35 additional plants from the Malvales order and Oxalidales order, as well as *Euphorbia tirucalli*, a member of the Euphorbiales order, as an outgroup. *Betula cordifolia* and *Quercus coccinea* which belongs to Fagales order to help to distinguish the boundaries. *S. sinensis* is closely related to *E. japonicus*. There is no doubt that they belong to Elaeocarpaceae family. Interestingly, base on traditional plant taxonomy which refers to the classification relationship obtained only by plant morphology without the help of molecular genetic information, Elaeocarpaceae family belongs to Malvales, and it belongs to Oxalidales in the taxonomy in NCBI. We found that the four Malvales families do not clearly crystallize out as expected, based on the alignment of their chloroplast genomes (Figure 1). The result of phylogenetic analysis may more support that *S. sinensis* is a member of Elaeocarpaceae family which belongs to Oxalidales order. To determine its evolutionary relationships more definitively, the chloroplasts genomes of more Elaeocarpaceae family members require sequencing.

**Figure 1.**
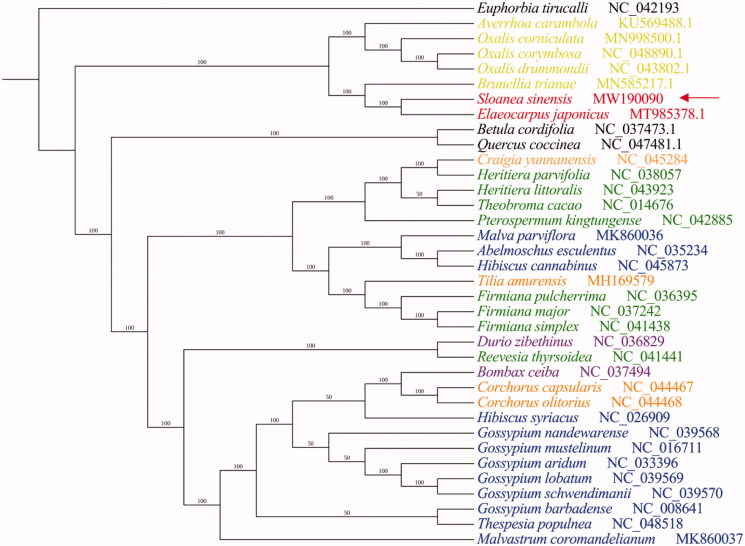
Bayesian inference (BI) generated from a complete chloroplast genome sequence alignment of 37 plant specie, using *Euphorbia tirucalli* as an outgroup. The number near the clade is posterior probability in percentage format. The Malvales order is subdivided in several plant families, indicated by color coding: the Malvaceae (blue), Tiliaceae (orange), Bombacaceae (purple), Sterculiaceae (green) and Elaeocarpaceae (red), as well as Oxalidales order (yellow).

## Data Availability

The genome sequence data that support the findings of this study are openly available in GenBank of NCBI at (https://www.ncbi.nlm.nih.gov/) under the accession no. MW190090. The associated BioProject, SRA, and Bio-Sample numbers are PRJNA685222, SRR13254435, and SAMN17080382, respectively.
